# Disproportionate Rates of Liver Injury in People With Cystic Fibrosis on Elexacaftor/Tezacaftor/Ivacaftor in Queensland, Australia

**DOI:** 10.1016/j.gastha.2025.100641

**Published:** 2025-02-08

**Authors:** Ellie Johnson, Timothy Riddles, Daniel Smith, Daniel Henderson, Philip Masel, David W. Reid, Vanessa Moore, Ieuan E.S. Evans

**Affiliations:** 1 Adult Cystic Fibrosis Centre, The Prince Charles Hospital, Brisbane, Queensland, Australia; 2 Faculty of Medicine, University of Queensland, Brisbane, Queensland, Australia; 3 Lung Inflammation & Infection, QIMR Berghofer Medical Research Institute, Brisbane, Queensland, Australia

The introduction of elexacaftor/tezacaftor/ivacaftor (ETI) has dramatically improved the overall health status of people with cystic fibrosis (pwCF). The two landmark studies of ETI tolerability found very low rates of cessation secondary to liver function test (LFT) derangement, 1% and 0%, respectively, although elevated aminotransferase levels were seen in 10.9% and 4%, respectively.^1^^,^^2^ Subsequent analyses of real-world rates of drug-induced liver injury associated with ETI showed an incidence of 2.1% in the Food and Drug Administration adverse event reporting system.^3^ Mild increases in both alanine aminotransferase (ALT) and aspartate transaminase (AST) not requiring alterations in treatment were also reported in a large single-center analysis from Manchester, UK.^4^ However, concerns about hepatotoxicity have led to “black box warnings” with subsequent changes to ETI safety labeling in both Brazil and the United States to include drug-induced liver injury and liver failure. While the mechanism resulting in hepatotoxicity secondary to ETI remains unclear, there have been isolated reports of hepatic necrosis with moderate mixed lobular inflammation on liver biopsies.^5^ It has been suggested that due to extensive metabolism via P450 CYP3A system, toxic or immunogenic metabolites of ETI may result in hepatotoxicity, though this remains unproven.^6^ We report on significantly higher rates of liver dysfunction necessitating ETI cessation or dose adjustment in a study of pwCF living in Queensland, Australia.

We conducted a prospective observational study of pwCF attending a state-wide adult cystic fibrosis (CF) service incorporating the Adult CF Centre at The Prince Charles Hospital and two large regional CF clinics in subtropical/tropical Queensland prescribed ETI between 1st January 2019 and 1st April 2024 (n = 281). Systematic analysis was conducted to ascertain the requirement for cessation and/or dose adjustment of ETI directly because of hepatotoxicity. Comparisons were then drawn against international clinical trial data to identify if distinct patterns existed in our cohort and explore the potential reasons for this. Ethical approval for the study was obtained from the Metro North Health Human Research Ethics Committee (HREC/2019/QPCH/46169).

Among the 281 pwCF prescribed ETI between 1st January 2019 and 1st April 2024, a total of 56 pwCF (19.9%) experienced dose adjustments or cessation of ETI for several different reasons including headaches, deteriorating mental health, and rash. Notably, 23 (8%) pwCF experienced hepatotoxicity felt to be directly related to ETI. Blood sampling following the initiation of ETI was conducted for all pwCF under the care of our CF center at one month, and, if no safety concerns were found, 3 monthly testing was initiated for the first year of treatment thereafter. Following this, annual blood testing was implemented as per current CF guidelines for standard monitoring. The median time to LFT derangement was 52 days (range 7–1440 days). Alterations in treatment were based on a combination of clinician discretion, considering aspects such as existing comorbidities and ETI product information, with cessation advised in the context of ALT and/or AST ≥5 times the upper limit of normal (ULN), or ≥3 times ULN with a bilirubin ≥2 times ULN.

**Figure 1:**
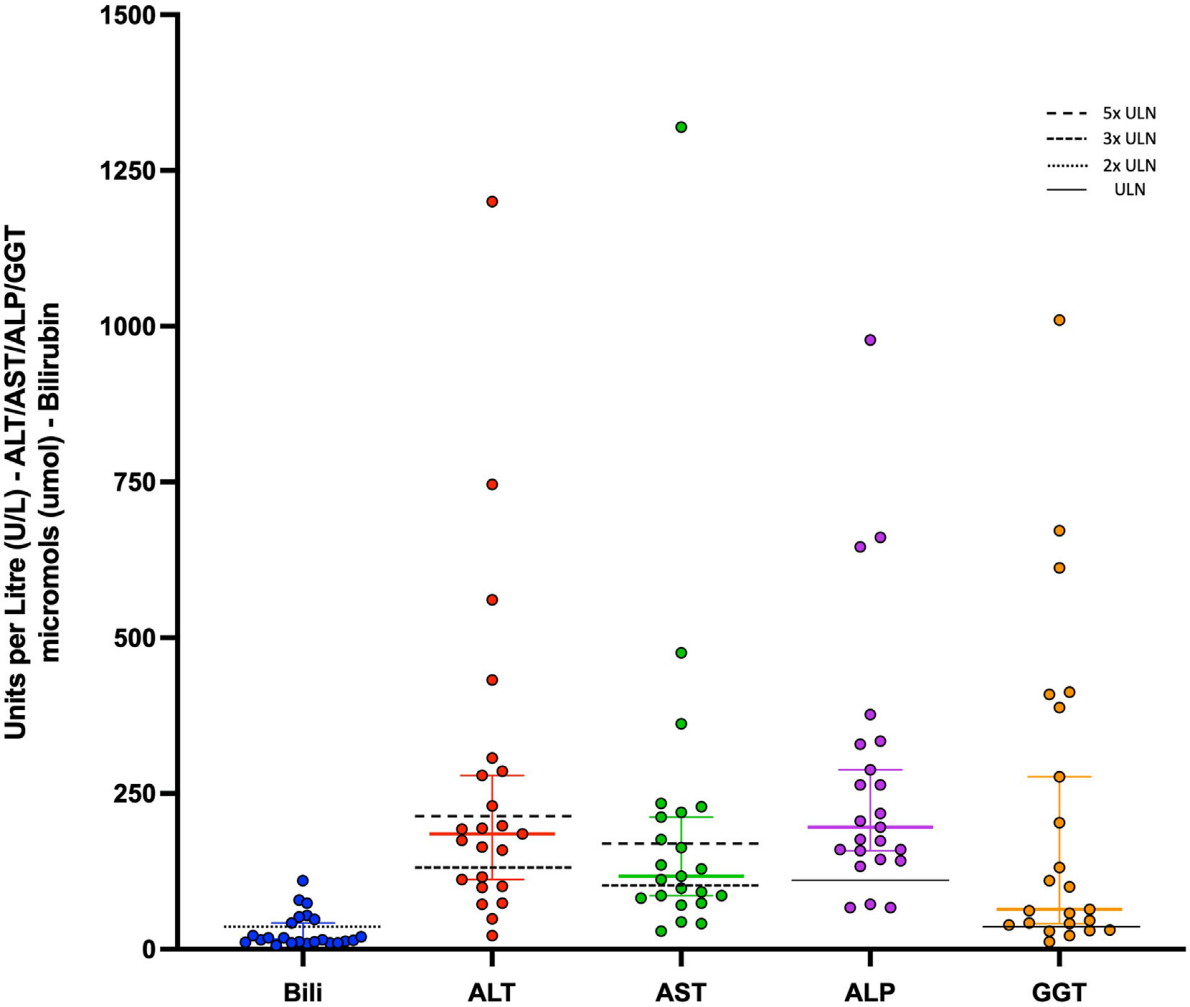
Peak ALT and AST levels in pwCF exhibiting hepatotoxicity directly related to ETI. ALP, alkaline phosphatase; Bili, bilirubin; GGT, gamma-glutamyl transferase.

Of the 23 pwCF noted to have ETI-associated hepatotoxicity, 15 experienced ALT peaks more than 3 times the ULN, with 8 reaching levels more than 5 times the ULN; 13 pwCF experienced AST peaks more than 3 times the ULN, with 8 experiencing levels more than 5 times the ULN; and 7 pwCF experienced bilirubin levels of more than 2 times the ULN. Of the 7 pwCF with elevated bilirubin levels, 5 had associated ALT or AST rises of 3 times the ULN (Figure 1). Only one pwCF was known to have issues with past or present alcohol excess; all were lifelong nonsmokers, and none were known to use any other nicotine inhalation devices such as electronic cigarettes. Underlying CF-related liver disease (CFLD) was present in 8 pwCF, with a further 2 having had liver transplants during pediatric care. Underlying CFLD had been confirmed on abdominal ultrasound scanning, with 5 pwCF exhibiting features consistent with cirrhosis and 4 with hepatic steatosis. Three of these pwCF had also undergone investigation with magnetic resonance cholangiopancreatography, one of which showed evidence of intra-hepatic biliary strictures.

**Figure 2:**
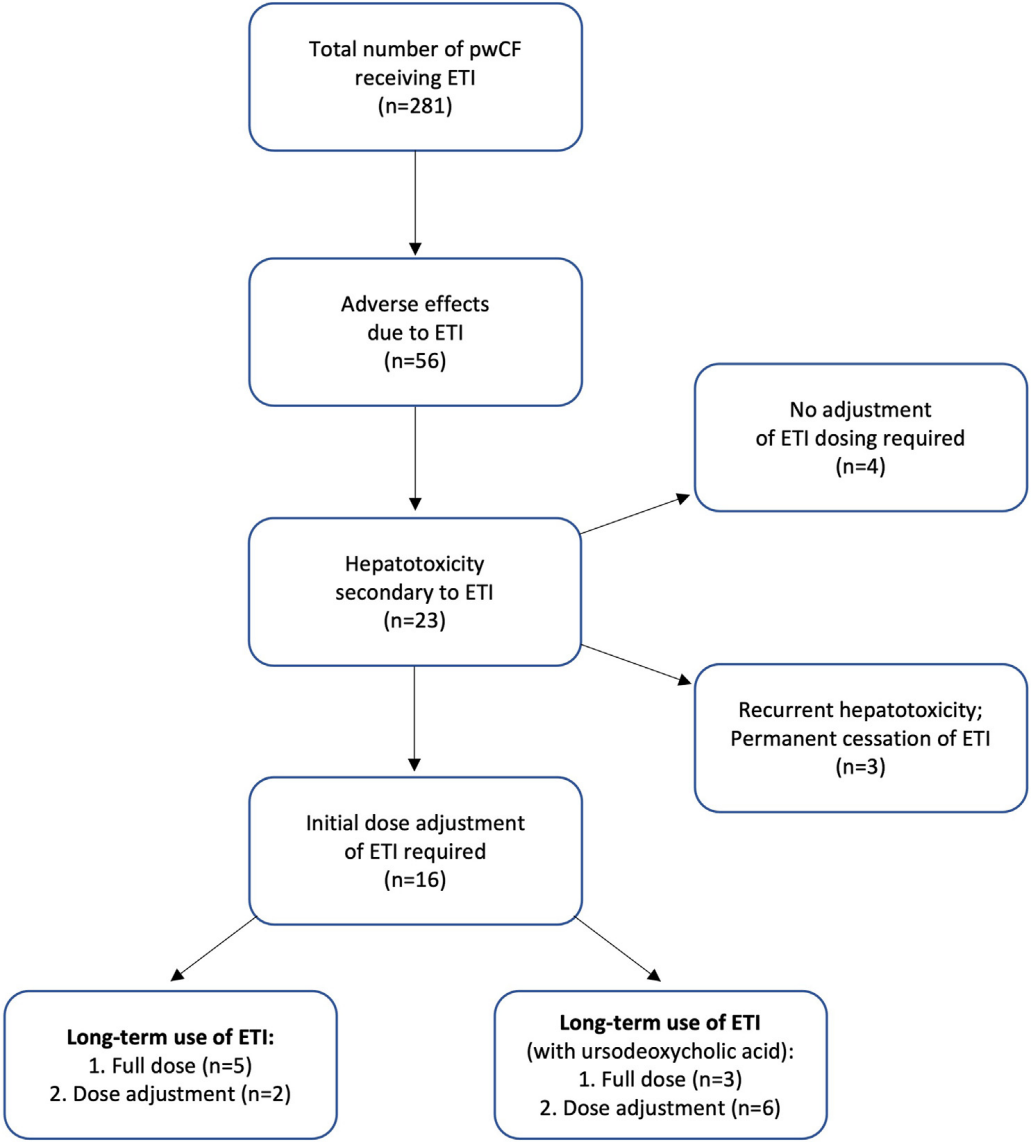
Flow chart highlighting treatment outcomes for pwCF exhibiting hepatotoxicity related to ETI.

Analyzing the 23 pwCF experiencing hepatotoxicity further, 19 required a break in treatment with subsequent reinitiation of ETI. However, 3 of the pwCF who reinitiated ETI experienced repeated episodes of significant hepatotoxicity resulting in permanent cessation of ETI therapy. In the 16 pwCF where ETI was successfully reintroduced, only 8 managed to tolerate a “full dose” following a gradual uptitration without further evidence of hepatotoxicity, with 3 of these pwCF commencing on ursodeoxycholic acid. The remaining 8 pwCF were able to tolerate a reduced dose with close monitoring of LFT results (Figure 2). Three pwCF were found to have ALT levels that peaked at 21, 22, and 35 times the ULN (respective AST levels of 7, 15, and 43 times ULN). Underlying CFLD was present in all 3, with one pwCF having had a previous liver transplant. The median time ETI was withheld before reintroduction was 68 days (range 6–459 days). No specific protocol was in place for the reintroduction or retrialing of ETI therapy. The decision to reintroduce ETI was based solely on the time taken for LFTs to sufficiently reduce to a level deemed safe for reinitiation of ETI. This was determined on an individual pwCF basis by the CF clinician responsible for management. Full hepatotoxicity screening was carried out for 15 (65.2%) pwCF to exclude other contributing pathologies and performed in all pwCF who had experienced an ALT rise ≥3 times ULN. Testing included blood screening for hepatitis A to E viruses, Epstein-Barr virus, cytomegalovirus, antinuclear antibody testing, extractable nuclear antigen antibodies panel, and immunoglobulins. All pwCF returned negative results.

As previously highlighted, attempts were made to ameliorate the chances of recurrent hepatotoxicity with the reintroduction of ETI. Nine pwCF were commenced on ursodeoxycholic acid (20 mg/kg/day); of these pwCF, 3 experienced no further issues with hepatotoxicity on reinitiation up to full dose, with the remaining 6 pwCF continuing reduced dose ETI. The decision regarding the introduction of ursodeoxycholic acid was made based on the degree of aminotransferase rise previously experienced and whether there was confirmation of underlying CFLD.

Published reports of a transient mild, clinically insignificant rise in aminotransferases seen on initiation of ETI differ from our local experience.^3^ This discrepancy suggests that, while ETI generally has a favorable safety profile, specific factors within our cohort may contribute to the increased hepatic dysfunction. One potential factor is the subtropical/tropical climate, with high environmental temperatures and humidity, leading to increased fluid and electrolyte losses. Frequent adjustments of fluid intake and electrolyte replacement are required depending on the season, and inadequate hydration is postulated to contribute toward an increase in liver dysfunction noted for pwCF on ETI. The environmental exposures in Queensland that may enhance dehydration are more common than experienced by pwCF in other published study populations, which may potentially explain the higher rates of liver dysfunction observed in our cohort. Analysis of this phenomenon will form the basis of ongoing research in our cohort with prospective data collection on hydration status, including urinary sodium/creatinine ratios for pwCF experiencing hepatotoxicity as part of further research. Other aspects being considered alongside this include variables such as age, gender, genetic predisposition, baseline and 12-month lung function, metabolic status, and preexisting CF-related liver disease. The addition of ursodeoxycholic acid appeared to aid the reintroduction of ETI in certain pwCF in our cohort. However, the impact of ursodeoxycholic acid used more widely in the setting of ETI-induced hepatotoxicity remains unclear and forms part of ongoing research. Future research directions should also focus on the biological mechanisms underlying liver dysfunctions in pwCF to better understand and mitigate the risks.

In summary, our study suggests that hepatotoxicity tends to occur early in ETI treatment, can recur upon reintroduction, and often requires dose adjustments or cessation of treatment. The utility of ursodeoxycholic acid in the setting of hepatotoxicity remains unclear. However, its use in the setting of ETI hepatotoxicity and underlying CFLD remains of interest and warrants further evaluation. While ETI is crucial for the management of CF, our findings highlight the need for heightened vigilance and tailored management, especially in warmer climates. Improved understanding of contributory factors will be essential for optimization of ETI dosing protocols and improved long-term outcomes. Furthermore, understanding the longer-term impact of hepatotoxicity secondary to ETI and whether low-grade hepatic inflammation in the setting of therapy leads to the development of structural changes within the liver, such as hepatic steatosis, remains a key unanswered question.
